# The Relationships between Gut Microbiota and Diabetes Mellitus, and Treatments for Diabetes Mellitus

**DOI:** 10.3390/biomedicines10020308

**Published:** 2022-01-28

**Authors:** Cristian-Ioan Craciun, Maria-Adriana Neag, Adrian Catinean, Andrei-Otto Mitre, Adriana Rusu, Cornelia Bala, Gabriela Roman, Anca-Dana Buzoianu, Dana-Maria Muntean, Anca-Elena Craciun

**Affiliations:** 1 Department of Pharmacology, Toxicology and Clinical Pharmacology, Iuliu Hatieganu University of Medicine and Pharmacy, 400337 Cluj-Napoca, Romania; cristian.craciun@umfcluj.ro (C.-I.C.); abuzoianu@umfcluj.ro (A.-D.B.); 2 Department of Internal Medicine, Iuliu Hatieganu University of Medicine and Pharmacy, 400006 Cluj-Napoca, Romania; catinean@gmail.com; 3 Faculty of Medicine, Iuliu Hatieganu University of Medicine and Pharmacy, 400012 Cluj-Napoca, Romania; andrei.otto.mitre@elearn.umfcluj.ro; 4 Department of Diabetes, Nutrition, Metabolic Diseases, Iuliu Hatieganu University of Medicine and Pharmacy, 400006 Cluj-Napoca, Romania; adriana.rusu@umfcluj.ro (A.R.); cbala@umfcluj.ro (C.B.); groman@umfcluj.ro (G.R.); anca.craciun@umfcluj.ro (A.-E.C.); 5 Department of Pharmaceutical Technology and Biopharmaceutics, Iuliu Hatieganu University of Medicine and Pharmacy, 400012 Cluj-Napoca, Romania; dana.muntean@umfcluj.ro

**Keywords:** microbiota, diabetes mellitus, inflammation, nutraceuticals, *Bacteroides*, *Firmicutes*

## Abstract

Diabetes mellitus is considered to be a global epidemic. The combination of genetic susceptibility and an unhealthy lifestyle is considered to be the main trigger of this metabolic disorder. Recently, there has been increased interest in the roles of gut microbiota as a new potential contributor to this epidemic. Research, in recent years, has contributed to an in-depth characterization of the human microbiome and its associations with various diseases, including metabolic diseases and diabetes mellitus. It is known that diet can change the composition of gut microbiota, but it is unclear how this, in turn, may influence metabolism. The main objective of this review is to evaluate the pathogenetic association between microbiota and diabetes and to explore any new therapeutic agents, including nutraceuticals that may modulate the microbiota. We also look at several mechanisms involved in this process. There is a clear, bidirectional relationship between microbiota and diabetes. Current treatments for diabetes influence microbiota in various ways, some beneficial, but others with still unclear effects. Microbiota-aimed treatments have seen no real-world significant effects on the progression of diabetes and its complications, with more studies needed in order to find a really beneficial agent.

## 1. Epidemiology and the Socioeconomic Impact of Diabetes Mellitus

Diabetes mellitus (DM) is a major health issue around the world. The International Diabetes Federation (IDF) Diabetes Atlas Ninth Edition [1] estimated that there were 463 million people living with diabetes in 2019, and predicted an increase of 51%, i.e., up to 700 million people, by 2045. The same source estimated that, in Europe, there were 59 million people diagnosed with DM in 2019, and predicted the smallest increase around the globe of 15% (up 68 million) by 2045.

Out of the total number of DM, around 90% of the cases are type 2 DM (T2DM), 5–10% are type 1 DM (T1DM), and 1–2% are other forms of DM. In 2019, 20.4 million (15.8%) live births occurred from mothers with some form of hyperglycemia (T1DM or T2DM prior to or with onset during pregnancy or gestational diabetes) [1].

The world’s economic burden related to DM was estimated to be around 760 billion USD in 2019, with an expected increase up to 825 billion USD by 2045 [1]. Regarding the cost in number of deaths, 4.2 million people aged 20 to 79 years died in 2019 due to diabetes and related complications (1 death/8 s) [1].

To have a better understanding of the global impact of diabetes, it is important to consider that the rising number of people living with DM might be driven by the increased number of new cases (due mainly to obesity and unhealthy lifestyle), but also by an increased life expectancy (due to better health care of people living with diabetes or people with other chronic diseases that live longer and develop DM during the ageing process) [1].

## 2. Microbiota and Their Roles in Diabetes Mellitus

It was 2000 years ago that Hippocrates, the “father of medicine”, said “all diseases begin in the gut” and, today, we experience the same feeling. There are many microbes in the human body and, collectively, they form the microbiota [2]. This “organ” includes all the microorganisms (bacteria, fungi, viruses, and parasites) in the human body (intestine, skin, and mucosa) [3]. Most of the microbiota are in the colon, although the microbiota in the skin, oral cavity, small intestine, or vagina cannot be neglected, since they have important roles in protecting our body [2]. In the intestine, the dominant phyla (approximately 90%) are represented by *Firmicutes* and *Bacteroidetes* with the genera *Lactobacillus*, *Bacillus*, *Clostridium*, *Enterococcus*, and *Ruminococcus* (of the first phylum) and *Bacteroides* and *Prevotella* (of the second phylum) [4,5].

Colonization of the body by microorganisms begins at birth and changes in the first year of life, affecting human health during its entire lifespan [6]. Microbiota can be negatively influenced by early life events such as cesarean section delivery, exposure to antibiotics, and artificial milk feeding [7]. Cesarean section and antibiotic therapy given to a mother seem to influence the maternal as well as the neonatal intestinal microbiota. In addition, the abundance of *Lactobacillus* and *Bifidobacterium* in breast milk decreases if a mother has been treated with antibiotics during pregnancy or breastfeeding [8].

Various factors can influence the microbiota, not only in infants but in early childhood. Changes in the composition and function of the microbiota can also develop in adults under the action of environmental factors, medical conditions (surgical events, certain diseases, and drug treatment), or living conditions (physical activity and smoking) [9].

Human microbiota play an important role in the homeostasis of the body. Changes in the composition and diversity of the microbiota can contribute to various diseases, ranging from gastrointestinal disorders, such as irritable bowel syndrome, and inflammatory bowel disease, to autoimmune diseases such as rheumatoid arthritis, systemic lupus erythematosus, and cancer [5,10,11,12]. In addition, a reduction in some metabolic changes caused by insulin resistance can hinder non-alcoholic fatty liver disease (NAFLD) development. The relationship between insulin resistance and NAFLD appears to be bidirectional [13]. The gut–liver axis has an important role in the development of NAFLD and metabolic syndrome. This axis represents the tight interaction between the gut microbiome and the liver [14]. The liver receives more than half of its blood supply from the splanchnic circulation, being most exposed to LPS and other microbiota-derived metabolites. This, together with the dysfunctional intestinal barrier increases the risk of development and progression of NAFLD, as well as other liver-related diseases [15].

Changes in the composition of the gut microbiota in patients with DM have been studied in recent years. The results have shown that the Firmicutes/Bacteroidetes ratio is higher in patients with diabetes as compared with healthy individuals. Moreover, certain bacterial species are increased or decreased in patients suffering from DM (Table 1).

Gut microbiota are involved in health and disease through: the synthesis of amino acids, the production of short chain fatty acids (SCFA), the absorption of nutrients, the prevention of colonization with pathological bacteria, the influence on the composition of bile acid, and the production of several pattern recognition molecules [24].

Amino acids play important roles in protein synthesis and metabolism and in signaling pathways. Among the bacteria with a high impact on amino acids are: the genus *Clostridium*, with a role in the fermentation of amino acids; *Peptostreptococcus*, with an impact on the use of glutamate and tryptophan; and bacteria from genera such as *Fusobacterium*, *Bacteroides* and *Veillonella* [25].

Dietary fibers, transformed into monosaccharides, colon proteins, together with amino acids resulting from intestinal fermentation of proteins in the large intestine are the main substrates for producing SCFA. Acetate, propionate, and butyrate are the main and most abundant SCFA [25,26]. Butyrate is mainly produced by the *Firmicutes* phylum, the family *Ruminococcaceae* (*Faecalibacterium prausnitzii*, *Clostridium leptum*), and the family *Lachnospiraceae* (*Eubacterium rectal*, *Roseburia* spp.) [27]. Acetate and propionate are produced by the *Bacteroidetes* phylum, i.e., acetate by *Bifidobacterium* spp., *Lactobacillus* spp., *Akkermansia muciniphila*, *Bacteroides* spp., *Ruminococcus* spp., *Prevotella* spp., and *Streptococcus* spp. and propionate by *Bacteroides* spp., *Ruminococcus obeum* and *Salmonella* spp. [28]. SCFA have important roles in regulating host metabolism and signaling pathways. For example, acetate is an important substrate for cholesterol synthesis; propionate increases high-density lipoprotein (HDL) cholesterol, improves insulin sensitivity, and glucose tolerance; and butyrate is the main source of energy for colonocytes, being involved in lipid metabolism [29,30]. All circulating SCFA have been shown to be positively correlated with glucagon-like peptide-1 (GLP-1), while they are negatively correlated with insulin sensitivity and free fatty acids. Only circulating butyrate has been shown to be negatively correlated with fasting plasma glucose [31].

Regarding the microbiota and bile acids, there is a two-way relationship. On the one hand, the microbiota can influence the composition of bile acids; on the other hand, bile acids have antimicrobial properties and can inhibit the overgrowth of bacteria. There are two types of bile acids: primary bile acids such as colic and chenodeoxycholic acids and secondary bile acids such as deoxycholic and lithocholic acids [32]. Primary bile acids, produced in the liver, are conjugated to glycine and taurine before entering the enterohepatic cycle. For the conjugation process, bile acids need to be hydroxylated to position 7. In this process, bacteria are involved such as *Bacteroides*, *Clostridium*, *Lactobacillus*, *Bifidobacterium*, and *Listeria*. Intestinal bacteria have the ability to convert primary bile acids to secondary bile acids by eliminating the hydroxyl group. The dehydroxylation process is performed under the action of *Clostridium* and *Eubacterium* [33,34]. Free and conjugated bile acids are ligands for FXR (farnesoid X receptor) and TGR5 (Takeda G-protein receptor 5) in the liver and intestine, and by activating them, the signaling pathways of metabolism, including glucose metabolism are influenced. Activation of FXR and TGR5 receptors causes certain pharmacological effects as follows: stimulates insulin secretion in pancreatic beta cells; stimulates glycogen synthesis via GSK3 (glycogen synthase kinase 3); increases GLP-1 secretion from L cells and thus, improves glucose metabolism and insulin resistance of the liver; signals to the central nervous system and modulates satiety; influences the NF-kB (nuclear factor-kappa B) pathway, which plays an essential role in inflammation; improves energy homeostasis; modulates thermogenesis [34,35,36,37].

It is known that DM is correlated with inflammation, a process with an important role in T1DM, T2DM pathophysiology, and their complications. Cellular and humoral immunity are involved in the pathogenesis of T1DM. It is believed that there are various factors that occur early in life, such as infections, nutrition, or the composition of the intestinal microbiota, and have the ability to contribute to the development of T1DM by activating immune cascades [38,39]. Proinflammatory mediators (IL-1 and TNF-α) are synthesized and released by stimulating innate pattern recognition receptors including toll-like receptors (TLRs) and retinoic acid-inducible gene 1 (RIG-I) [39]. TLRs interfere with inflammatory pathways and lead to an increase in proinflammatory cytokines [40]. Inflammation is an important etiological factor for insulin resistance, which may lead to the development of T2DM [41]. Insulin resistance also involves innate and adaptive immunity [42]. In addition, GPR43, one of the two orphaned coupled G protein receptors, appears to be important in intestinal immunity. This receptor is also recognized as FFAR. The main activators of GPR43 are acetate and propionate, and the consequences of these activations include: decreased cAMP, activation of the ERK pathway, increase in intracellular calcium, and activation of MAPK [43].

Correlations between proinflammatory cytokines and certain bacteria in the gut microbiota have been demonstrated: high TNF-α levels with decreased *Proteobacteria* and *Clostridiaceae*, increased *Bacteroidetes* spp., and decreased *Roseburia* spp.; IL-6 level with increased *Bacteroidetes* spp. and decreased *Roseburia* spp.; IL-1β level with an increase in *Bacteroides* spp. and *Veillonella* spp. and a decrease in *Bifidobacterium* spp., *Faecalibacterium* spp., and *Roseburia* spp. [44]. TNF-α can activate important pathways, such as intracellular IκB kinase, NF-kB, and JNK. TNF-α production in the pancreatic islets may disrupt normal insulin secretion in beta cells and may influence insulin resistance in several tissues (adipocytes, heart and skeletal muscle), in which TNF-α decreases the expression of glucose transporter type 4 (GLUT4) [45,46].

In healthy patients with healthy intestinal epithelium, LPS does not cross the intestinal barrier. Therefore, the maximum concentration of LPS occurs in the intestinal lumen and it is almost undetectable in the circulating plasma [47]. This effect is a consequence of the absorption of endotoxins from the gastrointestinal tract. The effect, called “metabolic endotoxemia”, is correlated with increased intestinal permeability, which is influenced by dysbiosis [48]. Intestinal permeability increases when tight junction integrity is affected. Thus, LPS enters the circulation and interacts with LPS-binding protein and membrane-bound CD14 receptor. Their complex interacts with TLR4, influencing both the inflammatory signaling pathways and the insulin signaling pathways [49,50]. TLR can be activated mainly by LPS, but also by endogenous products. An increase in circulating endotoxins that activate TLRs has been observed in both, T1DM and T2DM [48].

The most important SCFA in preventing increased intestinal permeability is butyrate. It plays an important role in mucin synthesis (increases mucin expression) and maintains tight junction integrity (interferes with junctional protein expression, i.e., zonula occludens protein-1 and occludin) [51,52].

## 3. Methods to Analyze Gut Microbiota

An accurate quantitative and qualitative measurement of gut microbiota related metabolites in biological samples is needed, in order to understand the relationships between these metabolites and the development of metabolic disorders.

Feces are a useful biological matrix that reflect the health of the lower gastrointestinal tract. The easiest and most straightforward approach appears to be the culture of fecal microbiota in selective, differential media. However, this method has proven to be unreliable since certain bacteria cannot be cultured. A large number of bacteria found in feces are strict anaerobes, with many of them being oxygen sensitive. As a result, during processing and culture, strict reducing conditions are required.

Mass spectrometry is a technique that is commonly used together with other techniques (gas or liquid chromatography) to assess metabolites such as fatty acids or bile acid products. However, there are some limitations in using these methods, i.e., many enzyme activities are not limited to a given microorganism or bacterial community [53].

Therefore, in recent years there has been rapid growth and improvement of analytical methods by using a derivatization step [54]. The aim of this derivatization is to transform the analytes into volatile molecules, thermally stable (in gas chromatography), and to provide species with better retention and ionization efficiency for non-volatile metabolites (in liquid chromatography). This derivatization step allows quantification of a large number of metabolites, with increased detectability, specificity, and chromatographic performance and reduced matrix effects, leading to high accuracy and precision of the assay [55,56].

A novel approach to bacterial enumeration using rRNA-molecule-targeting RT-qPCR seems to be very helpful in quantifying the dynamic changes occurring in the gut microbiota during host aging [57].

Therefore, feces are a dynamic and diverse matrix. Despite all the research, there is still a lot of work to be done on this subject, both in the analytical field to develop an optimal method to determine GM-related metabolites, and in the biological field to look for a link between gut bacteria and health or disease status.

## 4. Microbiota and Therapy for Diabetes Mellitus

The management of DM has the potential to be modulated by interaction with gut microbiota. Lifestyle interventions (especially diet) and pharmacotherapy (especially oral agents) used to control hyperglycemia can influence or be influenced by the microbiome constellation. In human studies performed in T2DM patients, it has been difficult to disentangle gut signature of the DM from those of a specific diet or medication [58]. Diet as a potential therapy in DM via microbiome is discussed in Section 5.

### 4.1. Metformin

Metformin is prescribed as first line pharmacotherapy in T2DM according to the guidelines recommendations, due to its good safety profile, good antihyperglycemic efficacy, pleiotropic effects and low cost [59]. Regarding its pharmacological effect, metformin reduces hepatic glucose production, but it has been shown that not all of its beneficial effects can be explained only by this mechanism of action and there is increasing evidence of pleiotropic effects, including direct action on the gut [23]. Many scientific data underline its promising beneficial effect on gut dysbiosis which is present in T2DM [60].

Metformin can act as a growth factor for some gut bacteria, such as *A. muciniphilla* (probably by metformin’s capacity to increase mucin-producing goblet cells in the intestinal wall, independent of diet) [60,61], and it also has the potential to significantly increase *Escherichia* and to lower *Intestinibacter* abundance [58]. Metformin-host microbiota interactions can explain the heterogeneity in glucose homeostasis and the intensity of side effects (bloating and intestinal discomfort) [58]. There is also scientific support for microbiota mediation of the therapeutic effects of metformin via enhanced production of gut peptides. Part of the therapeutic and intestinal side effects of the metformin can be explained by the increased secretion of GLP-1 from L-cells, and less by the decreased action of DDP-4 enzyme [62,63]. Other potential beneficial effects of metformin via gut microbiota can be explained by improved intestinal glucose sensing and by regulating gut permeability [58,60].

In experimental studies on rats fed a high-fat diet, metformin has been shown to decrease the bacterial diversity [64]. Metformin treatment in rodents fed a high-fat diet resulted in an increase in *A. muciniphila* and a decrease in *Clostridium orbiscindens*. Furthermore, *A. muciniphila* has been shown to be negatively correlated with serum glucose, while *Clostridium orbiscindens* has been negatively correlated with PPARα and GLUT2 and has been positively correlated with TNF-α, MUC2, and MUC5 [65]. An animal study has also proven that the intestine is the main site of metformin-associated lactate production [66]. Metformin can modulate SCFA production by increasing the population of *Blautia*, *Bacteroides*, *Butyricoccus*, or *Phascolarctobacterium* [61].

A clinical study realized on Japanese patients with T2DM showed that small changes in gut microbiota composition were observed after taking metformin for four weeks; a reduction in the Firmicutes/Bacteroidetes ratio was observed. This decrease was based on a reduced level of the Firmicutes phylum, and an increased level of the Bacteroidetes phylum [67].

The effect of metformin on gut microbiota, especially regarding the genus Lactobacillus, are not consistent between animal and human studies [68]. The explanation for confounding factors is that, in human studies, it is difficult to distinguish between innate bacteria and bacteria ingested with food and, therefore, the analysis of bacterial 16S rDNA has an important limitation because it cannot discriminate if the targeted bacteria are alive or dead [69].

### 4.2. Sulfonylureas

These drugs have hypoglycemic effects by closing ATP-sensitive K-channels in the pancreatic beta cell membrane, followed by insulin release in the blood stream [70].

In experimental studies, probiotic pretreatment with a mixture of *Bifidobacterium lactis*, *Lactobacillus acidophilus*, and *Lactobacillus rhamnosus*, in a study with alloxan-induced diabetes in rats, showed an elevated bioavailability of gliclazide [71].

In a recently published study using the parallel artificial membrane permeability assay (PAMPA) method, it was observed that probiotic bacteria and bile acids had important roles in bioavailability and patients’ responses to gliclazide, i.e., probiotic bacteria increasing the permeability of the drug [72].

On the one hand, in the urine of patients with T2DM treated with sulfonylureas, increased levels of hippurate and aromatic amino acids (phenylalanine and tryptophane) were observed, providing indirect evidence of the potential effect of sulfonylureas on the gut microbiota and its ability to process plant phenols and aromatic amino acids [73].

On the other hand, a recently published study showed that 12 weeks of treatment with gliclazide in T2DM patients had no impact on diversity and the composition of gut microbiota, and there were no significant associations between microbiome composition and clinical parameters [74].

### 4.3. Thiazolidinediones

Thiazolidinediones (TZDs) or ”glitazones” improve glycemic control in patients with T2DM by improving insulin sensitivity [75].

In experimental studies, pioglitazone was reported to decrease the alpha diversity and to shift the beta diversity of gut microbiota in C57BL/6J mice [76]. In a study with obese rats (Sprague–Dawley rats, rattus norregicus) after 8 weeks of high-fat diet, pioglitazone had a partial beneficial effect by reducing the abundance of *Proteobacteria*, but had no influence on *Enterobacteriaceae* and *Desulfovibrionaceae* [77]. In a rodent study, after only thirty days of high-fat diet, an increase in *Firmicutes*, *Proteobacteria*, and *Verrucomicrobia*, and a decrease in *Bacteroidetes* and *Candidatus arthromitus* were observed. Those modifications were reversible after starting a normal diet or one week treatment with rosiglitazone [78]. In a recently published study, diabetic B6.BKS(D)-Lepr^db^/J (db/db) mice that received rosiglitazone for 8 weeks developed changes in segment-specific host gene expression, without modifications of gut bacterial composition [79]. These data suggest a possible involvement of the TZDs on microbiota, but human studies regarding the impact of the drug on the microbiota in T2DM have not been reported.

### 4.4. Alpha-Glucosidase Inhibitors

Acarbose has a therapeutic effect in DM by inhibiting α-glucosidase, resulting in a reduction in the conversion of oligosaccharides into mono- and disaccharides and delaying postprandial intestinal glucose absorption. By its mechanism of action, it plays a role in providing nutrients to gut bacterial population. An older study showed that acarbose could also increase colonic butyrate production by a mechanism involving an increase in the concentrations of starch-fermenting and butyrate-producing bacteria [80].

Acarbose has also been recently indicated to have the potential to modify microbiota composition in people with prediabetes and T2DM. In a crossover trial with patients diagnosed with prediabetes, acarbose administration was followed by a modification of intestinal microbiome, meaning a reduction in *Butyricicoccus*, *Phascolarctobacterium*, and *Ruminococcus* and an increase in *Lactobacillus*, *Faecalibacterium*, and *Dialister* [81]. In T2DM patients, acarbose proved to have the potential to increase the intestinal population of *Bifidobacterium longum* [82]. Acarbose modifies the spectrum of intestinal bacteria involved in the metabolism of bile acids, by increasing *Lactobacillus* and *Bifidobacterium* and depleting *Bacteroides*. T2DM patients with an abundance of *Bacteriodes* treated with acarbose had more changes in plasma bile acids and greater improvement in metabolic parameters than T2DM patients that had gut microbiota abundant in *Prevotella*. This findings might be used to stratify the T2DM population based on their gut microbiota composition prior to treatment and to identify those patients that may have more beneficial effects after administration of acarbose [83]. To date, it is not well understood to what extent these changes in gut microbiome can explain the therapeutic or side effects of acarbose [76].

### 4.5. DPP-4 Inhibitors

Dipeptidyl-peptidase IV (DPP-4) inhibitors have an indirect effect on glucose metabolism by inhibiting the degradation of the incretins, glucagon-like peptide-1 (GLP-1) [84]. In experimental studies in rats, sitagliptin and vildagliptin were observed to have the potential to reduce the diversity of microbiota, and to increase the SCFA-producing bacteria (*Blautia*, *Roseburia*, *Clostridium*, and *Baceroides*, *Erysipelotrichaeae*, respectively) and to correct the gut Firmicutes/Bacteroidetes ratio [85,86]. Interestingly, saxagliptin, produced an opposite effect on gut microbiota in mice (increased Firmicutes abundance), but it cannot be excluded that these results might have been driven by the different animal models (mice vs. rats). In addition, an obesity-related phylotype, the genus *Candidatus Arthromitus*, was significantly reduced after treatment with saxagliptin [87]. In an experimental study in mice, vildagliptin had an impact on the composition of the gut microbiota and also on their activity. Thus, this drug increased *Lactobacillus* spp. and propionate and decreased *Oscillibacter* spp. and toll-like receptor ligands 2 and 4 [88].

The results of rodent studies differ from those obtained in human clinical trials. A clinical study published by Smits et al. analyzed the fecal microbiota in patients with DM2 after 12 weeks of treatment with either sitagliptin or liraglutide. These drugs had been added to the previous treatment with metformin and/or sulphonylurea. The results showed that the added treatment had no significant effect on the composition of the fecal microbiota [89].

### 4.6. GLP-1 Receptor Agonists

GLP-1 receptor agonists (GLP-1 RAs) are injected subcutaneously or are administered orally.

In animal studies, modification of gut microbiota was observed, probably due to the fact that GLP-1 RAs modify gastric emptying (with potential effect on local pH and nutrient composition) and gut transit time, altering the intestinal environment and microbiota composition [61]. In a mice study, liraglutide administration was shown to substantially decrease the relative abundance of all obesity-related phylotypes. Liraglutide also modulates gut microbiota by increasing the gut expression of genera *Allobaculum*, *Turicibacter*, *Anaerostipes*, *Blautia*, *Lactobacillus*, *Butyricimonas*, and *Desulfovibrio* and reducing the abundance of the order *Clostridiales* (phylum *Firmicutes*) and *Bacteroidales* (phylum *Bacteroidetes*) [87]. Similar results were observed by Madsen et al. in diet-induced obese mice treated with liraglutide. There was a decrease in the abundance of *Firmicutes* (*Lachnospiraceae* and *Clostridiales*) and an increase in the abundance of *Proteobacteria* and *Verrucomicrobia* (e.g., *Akkermansia muciniphila*) or *Firmicutes* (*Clostridiales*) [90].

In another experimental study, an enhancement of SCFA-producing bacteria (*Bacteroides*, *Lachnospiraceae*, and *Bifidobacterium*) was observed [91]. The weight-loss effect of liraglutide can be partly explained by the modulation of the gut microbiota composition, leading to a more lean-related microbiome profile [87]. In addition, in an animal study, exendin-4 induced significant changes in eleven bacterial species [92].

The impact of GLP-1RA (liraglutide and dulaglutide) on gut microbiota has been evaluated in clinical trials in patients with DM2. A significant difference in the composition of the gut microbiota was observed between respondents and non-responders to GLP-1RA treatment. Thus, in the GLP-1RAs respondent group, the decrease in blood glucose level was positively correlated with *Bacteroides dorei*, *Roseburia inulinivorans*, *Lachnoclostridium* sp., and *Butyricicoccus*, while it was negatively correlated with *Prevotella copri*, *Ruminococcaceae* sp., *Bacteroidales* sp., *Dialister succinatiphilus*, and *Alistipes obese* [93].

### 4.7. SGLT2 Inhibitors

Sodium-glucose co-transporter 2 (SGLT2) inhibitors are a new class of oral antidiabetic drugs. An anti-hyperglycemic effect is exerted by inhibiting SGLT2 in the proximal convoluted tubule in order to prevent reabsorption of glucose and facilitate its excretion in urine [94].

The scientific data about the potential effect of SGLT2 inhibitors (SGLT-2i) on gut microbiota are limited. One animal study showed that dapagliflozin treatment caused subtle changes in the richness and diversity of microbiota in diabetic mice, with minor effects on the microbiota in a control group and that the Firmicutes/Bacteroidetes ratio was reduced in dapagliflozin-treated diabetic mice as compared with the other groups. It remains unclear what could be the relevance of these changes to treatment efficacy [95]. In another experimental study in rats with type 2 diabetes, dapagliflozin increased *Ruminococcaceae* and *Proteobacteria* (especially *Desulfovibrionaceae*), but *Lactobacillaceae* and *Bifidobacteriaceae*, two important beneficial bacteria, did not increase after this treatment [96].

A recently published study proved that 12 weeks of treatment with dapagliflozin had no impact on the diversity and composition of gut microbiota in T2DM patients and there was no significant association between microbiome composition and clinical parameters [74].

Sotagliflozin is a newly developed drug, being the first dual SGLT inhibitor. By inhibiting SGLT1 present in the gut, sotagliflozin increases the concentration of glucose in the colon where it could promote changes in microbiota and increase production of SCFAs [97]. In experimental studies, potent dual SGLT1/2 inhibition was observed to have minimum impact on luminal microbiota in chow-fed rodents [98]. However, in the same study, there was an impact observed on the Firmicutes/Bacteroidetes ratio when the animals were fed with a high-sucrose diet.

### 4.8. Insulin

Insulin has been saving the lives of patients with T1DM since 1922, and is also used in the therapy of T2DM to improve glycemic control. Despite a century of use in human therapy, there are little data about its effect on gut microbiota. Due to its route of administration by subcutaneous injection, it is not expected to have a significant effect on gut microbiota.

In an experimental study, it was observed that the administration of oral porcine insulin did not alter gut microbiota composition of NOD mice [99]. This lack of effect can also be explained by the low bioavailability in the gut of insulin administered orally, due to its denaturation by gastric acid. The objective, of another recently published experimental study, was to investigate the effects of metformin on gut microbiota and compared it with insulin treatment in rats with type 2 diabetes mellitus (T2DM). The effect of insulin on gut microbiota in rats with T2DM was modest, only three genera were changed in the insulin treatment group as compared with the metformin group, where the relative abundances of 13 genera were significantly changed. In the insulin treated group, an increased abundance of *norank_f_Bacteroidales_S24-7*_group and a decreased abundance of *Lactobacillus* and *unclassified_f_Peptostreptococcaceae* were observed [100].

Data about direct effects of insulin therapy on human microbiota are not yet available, but there is evidence that gut dysbiosis plays a role in the response to insulin therapy in T2DM. In a recently published study including 480 hospitalized patients (aged 65–95 years) with newly diagnosed T2DM and 180 healthy subjects without glucose metabolism abnormalities, IL-37 was observed to have a protective role in the elderly T2DM patients and sensitized them to insulin therapy through suppressing the gut microbiota dysbiosis. The expression of IL-37 was negatively correlated with the Bacteroidetes/Firmicutes ratio [101].

## 5. Gut Microbiota as Therapeutic Target in Diabetes Mellitus

Gut microbiota seem to be promising targets to treat DM [102]. Modification of intestinal microbiome can also be used as a tool in the prevention of DM, and the efforts to reduce the global burden of chronic diseases [103]. An interesting human study used metagenomic clusters to identify, in a pool of patients with prediabetes, those with T2DM-like or normoglycemic-like metabolism according to their fecal microbiome, therefore, a potential tool for the stratification of risk of developing diabetes in people with prediabetes [19].

### 5.1. Changes in Lifestyle

One of the most powerful therapies for treating obesity and related diseases, such as T2DM, is lifestyle optimization, namely healthy diet and daily physical activity.

#### 5.1.1. Healthy Diet

Diet is a major factor that promotes gut microbiota composition. Animal studies have shown that variations in gut microbiota structure can be explained in a proportion of 57% by dietary changes and only in a proportion of 12% by host genetic mutation [104]. In the same study, it was observed that, in high-fat diet groups, *Bifidobacterium* spp., which play important gut barrier-protecting roles, were nearly absent in all animals [104]. A human study, comparing the microbiota of European children with that of African children from Burkina Faso (with plant-based high-fiber diet), found significant differences between the groups. The microbiota of African children showed good representation of *Bacteroidetes* (from the genus *Prevotella* and *Xylanibacter*, with a special capacity to hydrolyze cellulose and xylan, which was absent in European children) and the amount of *Firmicutes* and *Enterobacteriaceae* (*Shigella* and *Escherichia*) were reduced. These changes in microbiota composition were associated with more SCFA in children with a plant-based diet [105]. The authors of the study also underlined the importance of preserving these special bacteria present in the microbiome of people with traditional lifestyles and plant-based diets, to maintain the biodiversity of human microbiota, especially from ancient communities, due to the potential negative effect of globalization. Regarding the relation between the risk of T2DM and changes in overall plant-based diet index and health plant-based diet index over four years, recent published data from the Nurses’ Health Study (NHS) showed that each 10% increment in scores was associated with a 7–9% lower risk for the disease [106].

Fiber content to not the only food that has the potential to modulate gut microbiota. Studies analyzing protein content of the diet showed that protein intake was positively associated with overall microbial diversity, but with notable differences between animal or plant sources. Proteins from plants were associated with an increase in *Bifidobacterium* and *Lactobacillus* and a decreases in *Bacteroides fragilis* and *Clostridium perfringens*, while animal proteins induced an increase in *Bacteroides*, *Alistipes*, and *Bilophila* [107]. In addition, the protein/carbohydrate ratio has been associated with modifications in gut microbiota. Diets with high protein/low carbohydrate intake are followed by low levels of *Roseburia* and *Eubacterium rectale*, and decreased levels of SCFA, that might be detrimental to colonic health [108].

A Mediterranean dietary pattern is recommended by the guidelines for the prevention and clinical management of DM [59]. Partially, the beneficial effect on health and disease might be mediated via changes in gut microbiome. In obese patients, the Mediterranean diet decreased the *Prevotella* and augmented the *Roseburia* and *Oscillospira* genera, changes that were accompanied by improved insulin sensitivity [109]. A recently published study showed that the Mediterranean diet modified the gut microbiome in old people and improved their health status (measured as different indexes of frailty, cognitive function, and inflammation). More than six hundred patients from five European countries (United Kingdom, France, Netherlands, Italy, and Poland) were included in the study, and after one year of dietary intervention, there was a positive modification in the bacterial population that produced short or branched chain fatty acids and the improvement was correlated with longer time and better adherence to the Mediterranean diet plan [110]. A recently published review, by Calabrese et al., concluded that there was a potential link between Mediterranean diet microbiota and the onset and progression of T1DM. The onset of T1DM might be modulated by the response of the immune system to high intestinal production of SCFAs, especially butyrate, and the maintenance of intestinal permeability promoted by the high fiber content of the diet. The Mediterranean dietary pattern may also be beneficial for better glycemic control in T1DM patients, with a decreasing risk of chronic complications [111].

A gluten-free diet modifies the abundance of intestinal bacteria in healthy patients, by increasing unclassified species of *Clostridiales* and *Lachnospiraceae* and decreasing *Bifidobacterium* (4 spp.), *Lachnospiraceae* (2 spp.), *Blautia*, *Dorea* (*longicatena* and another sp.), *E. hallii*, and *A. hadrus* [112]. In patients with active gastro-intestinal symptoms of celiac disease, a gluten-free diet promotes the growth of *Proteobacteria* and inhibits the growth of *Bacteroidetes* and *Firmicutes*, whereas in patients without symptoms, it modifies *Bifidobacteria* count per gram of feces [112].

Intermittent fasting is a dietary pattern very popular among adults around the globe. There are several types of intermittent fasting behaviors, among which is included the religious fasting, Ramadan. During the holy month of Ramadan, people fast from sunrise to sunset, eating a large meal after sunset and a light meal before sunrise. It was observed that, at the end of the holy month, there was an increase in the abundance of *Akkermansia muciniphilia* (known as a signature of metabolic health, a modification that was observed to be promoted by the treatment with metformin), but this change was not persistent after returning to a regular dietary pattern [113].

Artificial sweeteners are present in the modern diet, especially in people with obesity and DM, in the effort to prevent high caloric intake and hyperglycemia. Suez, J. et al. showed, in animal studies, that saccharin caused a dysbiosis (relative abundance of the *Bacteroides* genus and *Clostridiales* order associated with a reduction in *Lactobacillus reuteri*) which induced glucose intolerance [114].

#### 5.1.2. Physical Activity

Exercise has an indirect effect on gut microbiota by regulating gut physiology and morphology. Low-intensity physical activity reduces the intestinal transit time, while prolonged exercise can increase gut permeability [115]. It is interesting that a non-ingestible factor can influence gut microbiota. A study performed on rats provided evidence that voluntary wheel-running exercise was followed by an increase in the cecal concentration of n-butyrate [116]. Another study, in mice, showed that exercise could prevent weight gain in high-fat fed animals. Physical activity produced modification in the Bacteroides/Firmicutes ratio and the changes were proportional to the total running distance [117]. In conclusion, exercise has the potential to modulate the effect of an unhealthy diet via changes in gut microbiota, but more evidence from human studies is needed.

### 5.2. Nutraceuticals Influencing Gut Microbiota

One of the most studied probiotics is *Lactobacillus casei*, which can increase the abundance of *Bacteroidetes* and reduce the abundance of *Firmicutes*, with the growth of *Bacteroides* and *Allobaculum*. It also increases butyrate and prevents the consequences of high-fat diets. All changes lead to an improved insulin sensitivity, oxidative state, and lipid profile [118]. Another species studied is *L. rhamnosus*, which can improve fasting blood glucose, glucose tolerance, and lipid profile and can decrease levels of free fatty acids and oxidative stress (SOD, catalase), TNF-α, and Il-6. Metabolic effects appear to be the consequence of downregulation of glucose-6-phosphatase expression in the liver of rats with streptozotocin-induced diabetes [119]. The effects of other pro-, pre-, and symbiotics are shown in Table 2.

## 6. Conclusions

Microbiota metabolites have a strong influence in DM, through their various relationships with the host metabolism and implications in several pathways. There is a bidirectional relationship between DM and gut microbiota, i.e., DM changes the composition of the microbiota, and the changed microbiota influence the pathophysiology of the disease. Current therapeutical approaches that aim at restoring the microbiota through various pharmacological and non-pharmacological therapies have seen mixed results. Most nutraceuticals seem to have a positive effect on DM symptoms and complications, but it is not yet clear which product is the better one and for which patients. Future clinical studies and meta-analyses of the current research are needed in order to successfully establish an efficient therapeutic agent.

## Figures and Tables

**Table 1:** Changes in the composition of the gut microbiota in patients with diabetes mellitus in clinical studies.

Type of Diabetes	Changes in Diabetes	References
Type 1 diabetes mellitus (children)	Increase in the number of *Clostridium*, *Bacteroides* and *Veillonella* Decrease in the number of *Lactobacillus*, *Bifidobacterium*, the *Blautia coccoides*/*Eubacterium rectale* group, and *Prevotella genus*	[16]
Type 1 diabetes mellitus	Decrease in the number of *Prevotella* and *Akkermansia* Increase in the number of *Actinobacteria*, *Bacteroidetes*, and *Proteobacteria*, *Lactoabcillus*, *Lactococcus*, *Bifidobacterium*, and *Streptococcus*	[17]
Type 2 diabetes mellitus	Decrease in the proportion of *Firmicutes* Increase in the proportion of *Bacteroidetes* and *Proteobacteria*	[18]
Type 2 diabetes mellitus	Increase in the abundance of four *Lactobacillus* species and decreases in the abundance of five *Clostridium* species Decrease in the abundance of *Roseburia* and *Faecalibacterium prausnitzii*	[19]
Type 2 diabetes mellitus	*Firmicutes*, *Actinobacteria*, positively correlated with fasting plasma glucose *Bacteroidetes*, *Proteobacteria*, negatively correlated with fasting plasma glucose	[20]
Type 2 diabetes mellitus	Increase in *Lactobacillus* No changes in *Prevotella* genus	[21]
Type 2 diabetes mellitus	Increase in *Faecalibacterium prausnitzii* No significant changes in *Bacteroides fragilis* and *Bifidobacterium longum*	[22]
Type 2 diabetes mellitus	Increase in *Blautia* and *Serratia genus* Decrease in *Verrucomicrobia phylum*	[23]

**Table 2:** Nutraceutical agents influencing the gut microbiota in experimental and clinical studies.

Nutraceutical Agent	Type of Clinical Study/Type of Diabetes Mellitus	Effects	References
Prebiotics			
Inulin	Experimental study (rats with streptozotocin-induced diabetes)	Decreased fasting blood glucose levels Increased serum GLP-1 level Decreased serum IL-6 level Decreased abundance of *Desulfovibrio* Increased *Lactobacillus*, *Lachnospiraceae*, *Phascolarctobacterium*, and *Bacteroides*	[120]
Prebiotic: oligofructose-enriched inulin	Clinical study/T1DM	Decreased intestinal permeability Increased *Bifidobacterium* and *Actinobacteria* Increased C-peptide	[121]
Probiotics			
*Lactobacillus* G15 and Q14, separated from Chinese traditional fermented diary food	Experimental study (rats with streptozotocin induced diabetes)	Improved glucose intolerance Reduced serum lipid levels Decrease IL-1β, IL-8, and IL-6 levels Increased the concentration of GLP-1 and PYY Reduced intestinal mucosal permeability	[122]
*Bifidobacterium* spp.	Experimental study (mice with streptozotocin induced diabetes)	Decreased blood glucose level Reduced MCP-1 and IL-6 mRNA levels in adipose tissue Increased the levels of IR-β, IRS-1, and Akt proteins	[123]
*Lactobacillus gasseri* BNR17	Experimental study (C57BL/KS/J db/db mice)	Decreased fasting and post-prandial blood glucose levels Decreased HbA1c	[124]
*Lactobacillus* spp.	Experimental study (rats with streptozotocin induced diabetes)	Decreased oxidative damage Inhibited the depletion of insulin	[125]
*Lactobacillus* spp.	Experimental study (high-fat diet mice)	Decreased fasting blood glucose levels No impact on insulin levels and lipid profile	[126]
*Lactobacillus plantarum*	Experimental study (rats with alloxan-induced diabetes)	Improves immunological parameters Protected the pancreatic, renal and hepatic tissues Reduced serum triglycerides and LDL cholesterol and increased the levels of HDL cholesterol	[127]
*Lactobacillus* spp.	Experimental study (rats with streptozotocin induced T1DM)	Improved glucose metabolism (HbA1c, fasting glucose, and insulin levels) Improved the inflammatory and oxidative stress status Improved the lipid profile	[128]
*Bifidobacterium* spp.	Experimental study (mice with streptozotocin induced diabetes)	Reduced blood glucose levels Decreased insulin resistance Induced adiponectin, MCP-1 and IL-6 expression	[123]
*Lactobacillus paracasei*	Experimental study (rats with streptozotocin-induced diabetes)	Decreased blood glucose levels, insulin resistance, and HbA1c Decreased glucagon and leptin levels Improved dyslipidemia and oxidative stress status	[129]
*Bifidobacterium bifidum*, *Lactobacillus casei*, *Lactobacillus acidophilus*	Clinical study (adults with T2DM)	Decreased fasting blood glucose, decreased HOMA-IR Increased HDL cholesterol levels Decreased serum hs-CRP Increased plasma TAC, increased GSH level	[130]
*L. reuteri* DSM 17938 (high dose)	Clinical study (adults with T2DM)	Improve the insulin sensitivity index Improved the serum deoxycholic acid Did not significantly improve HbA1c	[131]
*Lactobacillus* spp.	Clinical study/T2DM	Decreased insulin resistance Decreased triglycerides, IL-6 and MDA levels	[132]
*Lactobacillus* spp. and *Bifidobacterium* spp.	Clinical study/T2DM	Decreased fasting blood glucose levels and HbA1c Improved antioxidant status No changes in insulin concentration	[133]
*Lactobacillus*, *Bifidobacterium*, *Lactococcus* and *Propionibacterium* spp.	Clinical study/T2DM	Decreased HbA1c Decreased TNF-α and IL-1β Decreased insulin resistance	[134]
*Lactobacillus* spp.	Clinical study/T2DM	Decreased HbA1c and serum cholesterol levels Decreased blood pressure Reduced IL-1β Increased *Bifidobacterium* spp.	[135]
*Lactobacillus* and *Bifidobacterium* spp.	Clinical study/T2DM	Decreased HbA1c Improved fasting insulin levels	[136]
*Lactobacillus rhamnosus*	Clinical study/GDM	Lowered the relative rates of GDM Significantly lowered the prevalence of GDM	[137]
Symbiotics			
Selenium enhanced *Bifidobacterium* spp.	Experimental study (mice with streptozotocin induced diabetes)	Reduced levels of fasting glucose, HbA1c, leptin, and insulin Improved glucose tolerance and lipid profile Protected against liver and pancreatic impairment	[138]
*Lactobacillus sporogenes*, inulin as prebiotic, beta-carotene	Clinical study/T2DM	Significantly decreased serum insulin, HOMA-IR, HOMA-B, serum triglycerides elevated plasma NO, and GSH levels	[139]
Lactobacillus, Bifidobacterium species, S. thermophilus, and fructo-oligosaccharide)	Clinical study/T2DM	Decreased fasting blood glucose Decreased hemoglobin A1c Did not significantly influence lipid profile	[140]
*Lactobacillus* spp., *Bifidobacterium* spp. and oligofructose shake	Clinical study/T2DM	No significant decrease in total cholesterol and triglycerides Increased HDL Decreased fasting blood glucose levels	[141]
*Lactobacillus* spp., *Bifidobacterium* spp. *Streptococcus* spp. and fructo-oligosaccharide	Clinical study/T2DM	Decreased fasting blood glucose levels Increased insulin levels Increased LDL levels Increased total GSH levels	[142]
Other Nutraceuticals			
Caffeic acid-rich fraction of *Prunella vulgaris* L.	Experimental study (mice with alloxan-induced diabetes)	Reduced blood glucose levels and HbA1c Improved antioxidant activity Increased insulin levels	[143]
*Thymus marshallianus*	Experimental study (rats with streptozotocin-induced diabetes)	Reduced blood glucose levels Improved oxidative stress status Improved neurological functions	[144]

Akt, protein kinase B; CRP, C-reactive protein; DM, diabetes mellitus; GDM, gestational diabetes mellitus; GSH, glutathione; GLP-1, glucagon-like peptide-1; HOMA-IR/-B, homeostatic model assessment-insulin resistance/beta-cell function; HbA1c, glycated hemoglobin; HDL, high-density lipoprotein; IL, interleukin; IR-β, insulin receptor β; IRS-1, Insulin receptor substrate 1; LDL, low-density lipoprotein; MCP-1, monocyte chemoattractant protein 1; MDA, malondialdehyde; NO, nitric oxide; PYY, peptide YY; TAC, total antioxidant capacity; T1DM, type 1 diabetes mellitus; T2DM, type 2 diabetes mellitus; TNF, tumor necrosis factor.

## Data Availability

Not applicable.
